# Immune Checkpoint Inhibitors in Hepatocellular Carcinoma: An Overview

**DOI:** 10.3390/ph14010003

**Published:** 2020-12-22

**Authors:** Diederick J. van Doorn, Robert Bart Takkenberg, Heinz-Josef Klümpen

**Affiliations:** 1 Department of Gastroenterology and Hepatology, Amsterdam UMC, University of Amsterdam, Meibergdreef 9, 1105 AZ Amsterdam, The Netherlands; d.j.vandoorn@amsterdamumc.nl (D.J.v.D.); r.b.takkenberg@amsterdamumc.nl (R.B.T.); 2 Department of Medical Oncology, Amsterdam UMC, Cancer Center Amsterdam, University of Amsterdam, Meibergdreef 9, 1105 AZ Amsterdam, The Netherlands

**Keywords:** hepatocellular carcinoma (HCC), immunotherapy, systemic therapy, checkpoint inhibition, outcome, toxicity, tolerability, CTLA-4, PD-1, PD-L1

## Abstract

Patients with hepatocellular carcinoma (HCC) face a common type of cancer, which is amongst the most deadly types of cancer worldwide. The therapeutic options range from curative resection or ablation to loco regional therapies in palliative setting and last but not least, systemic treatment. The latter group underwent major changes in the last decade and a half. Since the introduction of sorafenib in 2007, many other systemic treatments have been investigated. Most without success. It took more than ten years before lenvatinib could be added as alternative first-line treatment option. Just recently a new form of systemic treatment, immunotherapy, entered the field of therapeutic options in patients with HCC. Immune checkpoint inhibitors are becoming the new standard of care in patients with HCC. Several reviews reported on the latest phase 1/2 studies and discussed the higher response rates and better tolerability when compared to current standard of care therapies. This review will focus on elaborating the working mechanism of these checkpoint inhibitors, give an elaborate update of the therapeutic agents that are currently available or under research, and will give an overview of the latest trials, as well as ongoing and upcoming trials.

## 1. Introduction

In 2018 hepatocellular carcinoma (HCC) was the sixth most common form of cancer and fourth most frequent cause of cancer-related death worldwide. Unfortunately, the incidence of HCC is constantly rising in the western world [1]. The etiology of HCC is mainly due to chronic or repeated habitual damage to healthy liver parenchyma resulting in the formation of fibrosis and eventually irreversible cirrhosis [2]. The underlying disease that causes liver cirrhosis varies from infectious diseases such as chronic hepatitis B or C virus infection to habitual damage due to excessive alcohol consumption or steatohepatitis [2,3,4,5,6]. The prevalence of diabetes type 2, together with obesity and cardiovascular diseases has risen over the past few decades and with this the prevalence of nonalcoholic fatty liver disease (NAFLD). The shift towards NAFLD as cause for liver cirrhosis is an important reason of increase of HCC-incidence in the western world [7].

Staging and treatment of HCC follows the Barcelona Clinic Liver Cancer (BCLC) staging, in which the disease is divided into (very) early stage (BCLC stage 0/A)—which is the only curative stage –, intermediate stage (BCLC stage B), advanced stage (BCLC stage C) and end stage disease (BCLC stage D) [8]. About 75% of the patients present themselves in a non-curative stage, leaving local treatment (BCLC stage B) and systemic treatment (BCLC stage C) as possible treatment modalities.

The world of systemic treatment has rapidly evolved over the past thirteen years. In 2007 sorafenib, a multi tyrosine kinase inhibitor (TKI), was introduced and has since then led the field of systemic therapy for over a decade [9]. In 2018 lenvatinib, a similar TKI, was registered as alternative for sorafenib in first-line treatment for HCC [10]. During this period, many other agents have been investigated. Most without success. However, for second-line therapy, regorafenib, cabozantinib and ramucirumab have been successfully added to the armamentarium of HCC therapy [11,12,13].

More recently, immunotherapy entered the field of HCC treatment [14]. Tumors use a wide variety of immunological escapes in order to survive. Even though these mechanisms are not understood completely, it provides the rationale for exploring immunotherapeutic options in cancer. One of the rationales behind immunotherapy in HCC relies mostly on the fact that the liver, as an organ, has to deal with many exogenous antigen exposures. For example, it has to perform detoxifying tasks for blood that has entered the portal circulation. Here, it has to deal with many antigen exposures. In order to prevent autoimmune damage, the liver developed intrinsic adaptive and protective mechanisms to cope with this immune response. Tumors of the liver seem to make use of these innate mechanisms to survive. In addition, since the etiology of HCC has mostly to do with chronic and constant inflammation, it triggers the hypotheses that the immune system plays an essential role in primary liver cancer [15].

At this time, the best understood mechanism is that of immune checkpoint inhibition. Tumors activate these immune checkpoints to incapacitate immune cells and their natural antitumor response. Hence, tumor growth depends on insufficient immunologic surveillance in the body. These immune checkpoints are proteins that inhibit immune cells and are vital for induction and maintaining tumor immune tolerance [16]. Since the introduction of immune checkpoint inhibitors in oncology in 2010 [17] and 2012 [18], promising results were found in a variety of cancers. Currently, inhibitors of cytotoxic T-lymphocyte-associated protein 4 (CTLA-4), programmed cell death protein 1 (PD-1) and its ligand (PD-L1) are well known and have been approved for several cancer treatments. Both CTLA-4 and PD-1 are proteins that are expressed as surface glycoproteins in T-lymphocytes. Once activated by their ligands, the T-cell activity gets inhibited and this can even lead to T-cell apoptosis. As a consequence, their antitumor immunity that is used to kill tumorous cells is prevented. Therefore, T-cells are logical targets for anticancer treatment [19].

Sadly, the first results from phase 3 trials aiming to test efficacy and safety of immune checkpoint monotherapy in HCC failed to show an increase in overall survival (OS) by not meeting its predefined significant threshold (see Section 2.1.1). However, other outcome variables such as tolerability were better compared to standard therapies, underlining the potential treatment benefit for HCC with immune checkpoint inhibitors.

On top of their therapeutic potential, immunotherapy seems to be well tolerated by patients in anticancer treatment when compared to, for example, chemotherapy [20]. Currently, multiple phase-3 trials are conducted to assess safety and efficacy of immune checkpoint inhibition in patients with HCC. Furthermore, to potentially increase the therapeutic effect of immunotherapy, combination therapies are also investigated. One of the most potent combinational therapy strategies in HCC seems to be an immune checkpoint inhibitor combined with a vascular endothelial growth factor (VEGF) inhibitor such as bevacizumab or IBI305 (a biosimilar). The rationale behind this combination is that HCCs are primarily arterially vascularized, in contrast to the rest of the liver, which gets its blood supply from the portal circulation. Inhibiting angiogenesis through anti-VEGF therapy leads to a halt in tumor growth and even induce shrinkage by cutting of the tumor’s blood supply. Another combination is with multiple immune checkpoint inhibitors itself and this has already shown to be more effective than monotherapy treatment alone in other types of cancers. However, whilst also increasing toxicity, combining immune checkpoint inhibitors is being increasingly investigated in clinical trials, of which the amount has doubled in the year 2020 [21,22]. Despite a more toxic profile, this nonetheless inspired researchers to explore the possibilities of combining two immune checkpoint inhibitors as combinational therapy in HCC [23].

Even though vaccination strategies are also a viable immunotherapeutic modality in the treatment or prevention of HCC, it will not be discussed in this review.

Over the past few years, many reviews have been published discussing the innovations in immunotherapy for patients with HCC [24,25]. Since then, new therapeutic agents have been registered and investigated and preliminary results from various studies have been published or presented at congresses such as ASCO or ESMO. The latest reviews focus mainly on elaborating the latest treatment strategies and their implications on the clinical field of HCC treatment [26,27]. This review will be an up-to-date addition to above-mentioned reviews. PubMed and ClinicalTrials.gov were searched until October 1th 2020.

This review aims to give an overview in the ever-developing field of immunotherapy in patients with HCC. It will do so by discussing the current therapeutic options, their working mechanism and to give a complete overview of completed, ongoing and coming trials with immunotherapeutic agents, including combination therapy.

## 2. Therapeutic Options

### 2.1. PD-1 and PD-L1 Pathway

The PD-1 protein is an immune co-inhibitory receptor, which is expressed on T and B cells and other immune cells. PD-1 sends out inhibitory signals when it interacts with its ligands PD-L1 and PD-L2. After binding, these ligands induce a two-way mechanism. In antigen-specific T-cells in lymph nodes, it will induce apoptosis (programmed cell death), while simultaneously reducing apoptosis in regulatory T-cells (Tregs), and reducing the functionality by blocking the T-cell receptor (TCR) and the signaling of CD28 [28]. In short: binding of PD-L1 with PD-1 of T-cell creates T-cell dysfunction, exhaustion and neutralization. PD-1 is expressed mainly on T-cells in a late stadium when coping with an infection or an inflammatory response. PD-L1 is expressed on a variety of tissues, such as blood vessels, myocardium and the lungs. Both PD-1 and PD-L1 are continuously being expressed at a constant rate in normal circumstances. This is one of the mechanisms the liver uses as a way to keep immune tolerance in a healthy liver. It is only when an immune response initiates, that PD-L1 is expressed on immune cells.

A majority of cancer cells in solid cancers also express PD-L1 on their surface and use this mechanism as a strategy to escape from immune surveillance. In HCC however, PD-L1 expression seems to be limited to a subset of particular HCC variants, making a majority of HCCs PD-L1 negative [29]. After recognition of cancer antigens by TCRs on activated T cells, molecules are released to attack these cancer cells. At the same time, cytokines are released which in their turn upregulate PD-L1 production of the cancer cells. This causes inhibition and exhaustion of the cytotoxic T lymphocytes and results in immune escape, as described above.

Therapy with anti-PD-1-antibodies nullifies this escape mechanism and allows T cells to remain in an active state despite the inhibitory strategy expressed by the tumor. The novelty aspect of this kind of treatment is that, unlike conventional chemotherapy and molecular targeted therapy, this treatment uses the body’s own immune system and its natural antitumor response and supports it.

**Figure 1:**
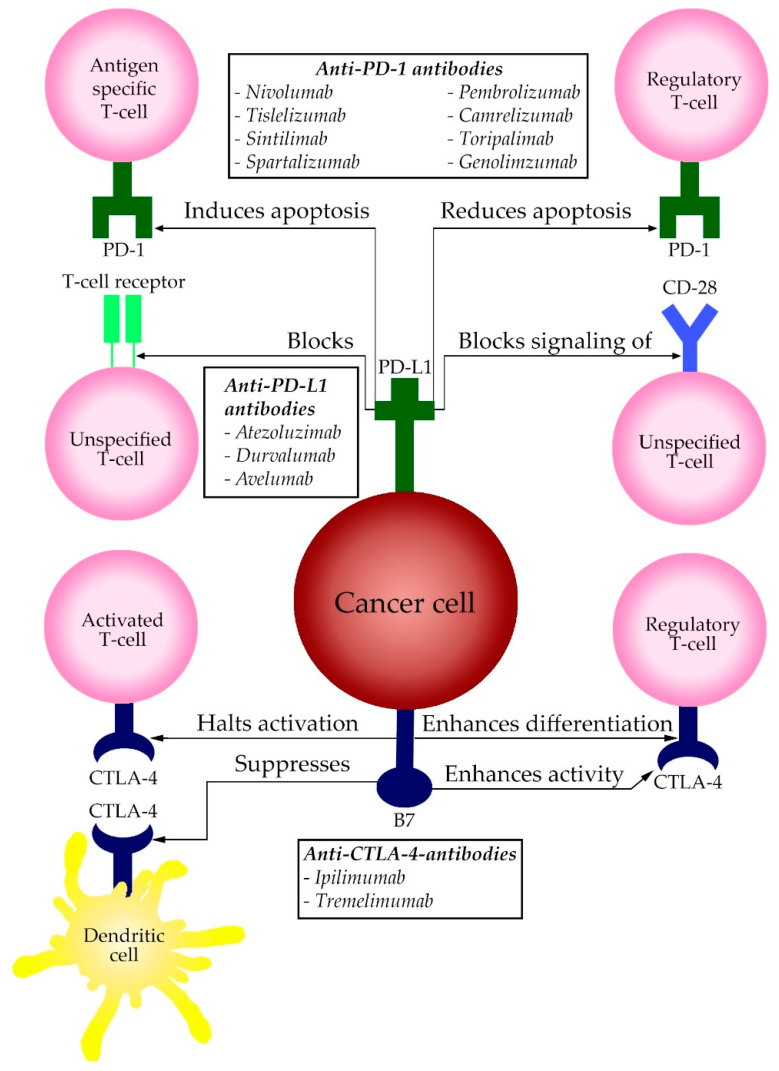
A schematic display of the PD-1, PD-L1 and CTLA-4 pathway and its effects on the human immune cells in the tumor microenvironment. The boxes display the therapeutic options clustered per site of action.

Anti-PD-L1-antibodies have an effect similar to that of PD-1-antibodies. PD-L1 overexpression is a marker of tumor aggressiveness [30]. PD-L1 is also being explored as a potential biomarker to predict the efficacy of anti-PD-1 therapy. Patients with high PD-L1 expression may be more likely to respond well to anti-PD-1 and anti-PD-L1 therapy. A schematic overview of the abovementioned pathway is displayed in Figure 1. All PD-1 and PD-L1 therapeutic agents that are approved for use in HCC and their most important publicized studies are summarized in Table 1.

#### 2.1.1. Nivolumab

Nivolumab is a human IgG4 monoclonal antibody that blocks PD-1 and is the first anti-PD-1-antibody for HCC approved by the U.S. Food and Drug Administration (FDA). It has been found effective as an alternative in second-line therapy for patients with HCC in the phase 1/2 open-label CheckMate040 trial [31].

The CheckMate040 trial showed promising results, and on these results alone, the FDA granted accelerated approval in September 2017. In this trial, a total of 214 patients received nivolumab and this was given to patients with hepatitis C and hepatitis B (HCV/HBV) and to patients who were already treated with sorafenib or were sorafenib naïve. The trial had a dose escalation and expansion component. Response to treatment was noteworthy. In total 42 (20%) of patients had objective response independently of prior treatment with sorafenib, including three complete responders. In this cohort 144 (67%) of patients had extrahepatic spread of disease and 63 (29%) patients had macro vascular involvement. Disease control rate was reached in 138 (64%) of patients. In total 48 patients discontinued treatment of whom 12 (25%) had grade 3/4 treatment-related adverse events (AEs). As a result, the phase 3 ChecMate459 trial [32] was initiated.

The CheckMate459 trial aimed to test the efficacy of nivolumab by testing it in first-line to show superiority over sorafenib. Unfortunately nivolumab failed to show a significant increase in median OS for its predefined statistical threshold (Hazard Ratio (HR) 0.84, *p* = 0.0419). In total, 743 patients were included and randomized into this study. For both the nivolumab and sorafenib arms however, OS was remarkably long, namely: 16.4 months and 14.7 months for nivolumab and sorafenib respectively (HR 0.85 [95% CI: 0.72–1.02]; *p* = 0.0752). Objective response rate (ORR) was 15% for nivolumab and 7% for sorafenib. A total of 14 (4%) patients reached complete response (CR) with nivolumab and 43 (12%) partial response (PR) versus 5 (1%) CR and 21 (6%) PR in sorafenib. Grade 3/4 treatment-related AEs were reported in 81 patients (22%) in the nivolumab arm and 179 patients (49%) in the sorafenib arm and led to discontinuation in 16 (4%) and 29 (8%) patients, respectively. Further analyses into OS and treatment benefit of nivolumab will follow.

#### 2.1.2. Pembrolizumab

Pembrolizumab is a humanized IgG4 monoclonal antibody and is the second anti-PD-1-antibody that has been approved for a variety of solid cancers and is currently under investigation for its use in HCC. The data of the KEYNOTE-224, a phase 2 clinical trial [33] and KEYNOTE-240, a phase 3 clinical trial [34] have been presented.

The KEYNOTE-224 trial was a non-randomized, multicenter, open-label, phase 2 trial that was set in 47 medical centers and hospitals across ten countries. Patients that were included were those with histologically confirmed HCC that were treated with sorafenib in the past without sufficient response. Of 169 patients screened, 104 received pembrolizumab every 3 weeks for about 2 years or until disease progression. Primary outcome of this study was objective response. ORR occurred in 18 (17%; 95% CI: 11–26) of 104 patients. The best overall responses were one (1%) complete and 17 (16%) partial responses. Forty-six (44%) patients had stable disease, and 34 (33%) had progressive disease. Treatment-related AEs occurred in 76 (73%) of 104 patients, which were serious in 16 (15%) patients. Grade 3 treatment-related AEs were reported in 25 (24%) of the 104 patients; the most common were increased aspartate aminotransferase concentration in seven (7%) patients, increased alanine aminotransferase concentration in four (4%) patients, and fatigue in four (4%) patients. One (1%) grade 4 treatment-related event of hyperbilirubinemia occurred. One death associated with ulcerative esophagitis was attributed to treatment. Immune-mediated hepatitis occurred in three (3%) patients, but there were no reported cases of viral flares.

The KEYNOTE-240 trial was a randomized, double blind, phase 3 study conducted at 119 medical centers in 27 countries. Patients included were those with advanced HCC, previously treated with sorafenib and were randomly assigned at a two-to-one ratio to receive pembrolizumab and best supportive care (BSC) or placebo with BSC. Primary endpoints were OS and progression free survival (PFS). Safety was assessed in all patients who received ≥ 1 dose of study drug. A total of 588 patients were screened for this study of whom 413 patients were randomly assigned. Median follow-up was 13.8 months for pembrolizumab and 10.6 months for placebo. Median OS was 13.9 months for pembrolizumab versus 10.6 months for placebo (HR, 0.781; 95% [CI: 0.611-0.998]; *p* = 0.0238). Median PFS for pembrolizumab 3.0 months versus 2.8 months for placebo at final analysis (HR, 0.718; 95% CI: 0.570–0.904; *p* = 0.0022). Although OS and PFS improved compared with placebo, they did not meet the pre-specified boundaries of *p* = 0.0174 for OS and *p* = 0.002 for PFS. ORR was 18% (95% CI: 14.0–23.4%) for pembrolizumab and 4% (95% CI: 1.6–9.4%) for placebo at final analysis with a nominal one-sided *p* = 0.00007. Best overall responses were six CRs (2%) and 45 PRs (16%). For the pembrolizumab group, 122 patients (44%) had stable disease (SD), and 90 (32%) progressive disease (PD). In the placebo group, there were no CRs; six patients (4%) had PRs, 66 (49%) had SD, and 57 (42%) had PD. Grade 3 or higher AEs occurred in 147 (53%) and 62 patients (46%) for pembrolizumab versus placebo. However, those that were treatment related occurred in 52 (19%) and 10 patients (8%), respectively. No hepatitis C or B flares were identified. The authors concluded that even though OS and PFS did not reach statistical significance per specified criteria. The results are consistent with those of KEYNOTE-224, supporting a favorable risk-to-benefit ratio for pembrolizumab in this population.

#### 2.1.3. Tislelizumab

Tislelizumab is a humanized IgG4 monoclonal antibody with high affinity and specificity for PD-1. It is currently being researched in a first-in-human study (NCT02407990). However, early reports and other early phase studies suggested tislelizumab was generally well tolerated and had antitumor activity in patients with advanced solid tumors. One report specifically looked into the effects of tislelizumab in patients with esophageal, gastric, hepatocellular, and non-small cell lung cancers [35]. Eligible patients received tislelizumab 2 or 5 mg/kg every 2 or 3 weeks. Median duration of study follow-up ranged from 4.9–9.9 months. Out of 207 patients included into these analyses, 50 were included with HCC diagnosis. ORR occurred in six (12%; 95% CI: 4.6–24.8) patients, 19 (38%) patients had stable disease and 23 (46%) had progressive disease. Treatment-related AEs in the total study cohort that occurred in more than 5% of patients were fatigue (8.7%), decreased appetite (6.8%), rash (6.8%), hypothyroidism (6.3%), and nausea (6.3%). Grade 3 and higher treatment related AEs occurred in 18% of patients and were pneumonitis (*n* = 3), elevated AST (*n* = 3), and elevated ALT (*n* = 2). Grade 5 treatment related AEs occurred in two patients: pneumonitis in a patient with non-small cell lung cancer (NSCLC) with compromised pulmonary function and acute hepatitis in a patient with HCC with rapidly progressing disease. The authors conclude that tislelizumab was generally well tolerated and antitumor activity was observed in each tumor type. Further research into tislelizumab as monotherapy or as combination, is currently being evaluated in multiple phase 2 and phase 3 studies.

#### 2.1.4. Camrelizumab (SHR-1210)

Camrelizumab is a humanized monoclonal anti-PD-1-antibody. It has been shown to block the binding of PD-L1 with a high affinity for PD-1. It recently has been globally approved for anti-cancer treatment [36]. Furthermore, it binds to epitopes different from nivolumab and pembrolizumab. Phase 1 clinical trials have shown that camrelizumab is well tolerated and has antitumor activity in patients with advanced solid tumors. The first results of a phase 2, open-label, parallel-group, randomized trial done at 13 study sites in China were recently published in the Lancet [37]. This trial aimed to assess the antitumor activity and safety of camrelizumab in second-line setting of patients with advanced HCC. In total 303 patients were screened for eligibility, of whom 220 patients were found eligible and were randomly assigned. A total of 217 patients actually received camrelizumab (109 patients were given treatment every 2 weeks and 108 patients every 3 weeks). Median follow-up was 12.5 months with an interquartile range of 5.7–15.5 months. ORR occurred in 32 (15%; 95% CI: 10.3–20.2) of 217 patients. The best overall responses were 0 CR and 32 (15%) PR. Stable disease was established in 64 (30%) of 217 patients.

The overall survival probability at 6 months was 74% (95% CI: 68.0–79.7) and the overall survival probability at 12 months was 56% (95% CI: 48.9–62.2). Grade 3/4 treatment-related AEs occurred in 47 (22%) of 217 patients; the most common were increased aspartate aminotransferase in ten (5%) and decreased neutrophil count in seven (3%) patients. Two deaths were judged by the investigators to be potentially treatment-related (one due to liver dysfunction and one due to multiple organ failure). Furthermore, this trial also looked into the expression of PD-L1. PD-L1 expression data were available in 30 patients. Objective response was achieved in four (36%) of 11 of patients with expression of PD-L1 1% or higher, whereas it was achieved in two (11%) of 19 patients with PD-L1 less than 1%. The authors concluded that the results suggested that camrelizumab has antitumor activity, a preliminary survival benefit, and a manageable safety profile, similar to other PD-1 immune checkpoint inhibitors. Therefore, they concluded that camrelizumab might be a potential second-line treatment strategy for patients with advanced HCC. Currently, multiple phase 3 trials assessing the efficacy as first-line therapy in advanced HCC are investigated as well as trials further exploring the second-line therapeutic potential for patients with HCC.

#### 2.1.5. Sintilimab (IBI308)

Sintilimab is a fully human IgG4 monoclonal antibody that binds to PD-1 that also has been recently approved for anti-cancer treatment [38]. It initially has been identified as treatment for classical Hodgkin’s lymphoma in the ORIENT-1 trial [39]. This was a single arm phase 2 study to assess activity and safety of sintilimab in patients with Hodgkin lymphoma. For patients with HCC, the ORIENT-32 trial is currently ongoing. The ORIENT-32 study is a randomized, open-label, multi-center, Phase 2/3 trial conducted in China to evaluate the safety and efficacy of sintilimab in combination with IBI305 (a recombinant humanized anti-VEGF monoclonal antibody), compared to sorafenib as first-line treatment in patients with advanced HCC. In total 566 patients are expected to be enrolled in the study. Interim analyses are expected any moment. This study follows a phase 1b study that evaluated sintilimab in patients with advanced HCC. Preliminary results of the phase 1b study were presented at the 2020 annual meeting of the American Society of Clinical Oncology [40]. Here, it showed promising results. As of January 7, 2020, a total of 50 patients were included, divided into a low-dose group (29 patients) and a high-dose group (21 patients). In the low-dose group, seven patients had confirmed PR, with ORR of 24%. In the high-dose group, six patients had PR with an ORR of 33%. Treatment-related AEs were mostly grade 1/2, with the most common including hypertension (28%) and pyrexia (26%); a total of 6 (12%) patients experienced grade ≥ 3 treatment related AEs, with the most common grade ≥ 3 AE being hypertension (2 patients). Altogether, results of the ORIENT-32 trial are awaited and could be promising.

#### 2.1.6. Toripalimab (JS001)

Toripalimab is a recombinant, humanized PD-1 monoclonal antibody that binds to PD-1 and prevents binding with its ligands. In December 2018, based on positive efficacy results of a phase 2 trial and safety data from several clinical studies, toripalimab received approval in China for the second-line treatment of unresectable or metastatic melanoma [41]. One well-known study is the POLARIS-01 study [42]. It was a phase 2, single-armed multi-center trial, which was designed to evaluate safety and efficacy of toripalimab in Chinese patients with advanced melanoma who had failed in previous systemic treatments. The primary objectives were safety and ORR. The study enrolled 128 melanoma patients. Among 127 patients assessed, one had CR, 21 had PR to treatment, and 51 had stable disease. Analysis of these results revealed an ORR of 17%. AE results were analyzed almost two years after last enrollment. In total 116 (90.6%) experienced treatment-related AEs. Grade ≥ 3 TRAEs occurred in 25 (19.5%) patients. For HCC there are no results yet available of the several clinical phase 2 and 3 trials are currently running.

#### 2.1.7. Atezolizumab

Atezolizumab is a fully human IgG1 monoclonal antibody against PD-L1 and is the first anti-PD-L1-antibody to be approved by the FDA for the treatment of various cancers. Atezolizumab was first studied in a safety and efficacy study in a single-arm clinical trial involving 310 patients with locally advanced or metastatic urothelial carcinoma [43]. In HCC, atezolizumab is best known for its combinatory treatment with bevacizumab, a monoclonal VEGF inhibitor. Bevacizumab demonstrated only a modest effect on HCC when given as single treatment. The rationale behind combining an immune-checkpoint inhibitor with an anti-VEGF antibody is that HCC is a hyper vascular tumor type where VEGF and PD-L1 are overexpressed.

The combination of atezolizumab and bevacizumab (AT-B) was first successfully tested in a phase 1b study (GO30140) assessing the efficacy of atezolizumab with or without bevacizumab in patients with unresectable HCC [44]. In the atezolizumab monotherapy group, 10 (17%; 95% CI: 8–29) of 59 patients had a confirmed objective response. CR occurred in three (5%) patients and PR in seven (12%) patients. Grade 3/4 treatment-related AEs occurred in three (5%) patients in the atezolizumab monotherapy group. In the AT-B group, 37 (36%; 95% CI: 26–46) of 104 patients had a confirmed objective response. CR occurred in 12 (12%) patients and PR in 25 (24%) patients. Grade 3/4 treatment-related AEs occurred in 44 (42%) patients in the AT-B group. The most common grade 3/4 treatment-related AEs were hypertension (15 (14%)) and proteinuria (seven (7%)). Treatment-related serious AEs occurred in 25 (24%) patients and treatment-related deaths in three (3%) patients (abnormal hepatic function, hepatic cirrhosis, and pneumonitis).

After this successful trial, the IMBrave150 trial was initiated and recently published its first preliminary results [45]. This trial was a global, open-label, phase 3 trial, including patients with unresectable hepatocellular carcinoma who had not previously received systemic treatment. In this, patients were randomly assigned in a 2:1 ratio to receive either atezolizumab plus bevacizumab or sorafenib until unacceptable toxic effects occurred or when there was a loss of clinical benefit. In total, 501 patients were included into study analyses. The intention-to-treat population included 336 patients in the atezolizumab-bevacizumab group and 165 patients in the sorafenib group. Results at the first primary analyses (29 August 2019) were promising. The HR for death with atezolizumab-bevacizumab as compared to sorafenib was 0.58 (95% CI: 0.42–0.79; *p* < 0.001). At 12 months OS was 67.2% (95% CI: 61.3–73.1) with atezolizumab-bevacizumab and 54.6% (95% CI: 45.2–64.0) with sorafenib. Median PFS was 6.8 months (95% CI: 5.7–8.3) for the atezolizumab-bevacizumab group and 4.3 months (95% CI: 4.0–5.6) in the sorafenib group (HR for disease progression or death, 0.59; 95% CI: 0.47–0.76; *p* < 0.001). Grade 3/4 AEs occurred in 56.5% of 329 patients who received at least one dose of AT-B and in 55.1% of 156 patients who received at least one dose of sorafenib. Grade 3/4 hypertension occurred in 15.2% of patients in the AT-B group. However, other high-grade toxic effects were infrequent. Altogether, the authors concluded that patients with unresectable hepatocellular carcinoma could benefit from AT-B, since it resulted in better overall and progression-free survival outcomes when compared to sorafenib.

#### 2.1.8. Spartalizumab (PDR001)

Spartalizumab is a humanized monoclonal antibody and is a relative new PD-1-antibody developed by Novartis (Basel, Switzerland). A recent first-in-human dose escalation study of the drug showed favorable toleration in patients who received the agent [46]. However, limited clinical activity was reported in the study population. ORR occurred in only two (3.4%) of 58 patients. The most common treatment-related AEs of any grade were fatigue (22%), diarrhea (17%), pruritus (14%), hypothyroidism (10%), and nausea (10%). Multiple phase 2 studies are currently ongoing in select tumor types. However, Novartis recently reported late-breaking data on the ESMO virtual congress 2020, that the phase 3 COMBI-i trial did not meet its primary endpoint of investigator-assessed PFS for patients treated with the investigational therapy [47].

#### 2.1.9. Genolimzumab

Genolimzumab is a humanized IgG4 monoclonal antibody against PD-1. It is currently still being investigated in a phase 1, first in human, multicenter, 3-part study with a dose-escalation segment, cohort extension and dose and disease expansion cohorts of genolimzumab injections in Australia in a variety of solid cancers (NCT03053466). Results are still awaited.

#### 2.1.10. Durvalumab

Durvalumab is, like atezolizumab, a human IgG1 monoclonal antibody against PD-L1. Previous studies reported a longer progression-free survival (PFS) with durvalumab as consolidation therapy compared with placebo in stage III NSCLC patients who did not have disease progression after two or more cycles of platinum-based chemo–radiotherapy [48]. Based on these data, durvalumab received FDA approval as consolidation chemotherapy in stage III NSCLC disease in February 2018. In HCC, durvalumab is currently studied as monotherapy or in combination with other agents. One phase 1/2, first-in-human study was conducted to evaluate the safety and clinical activity of durvalumab in patients with advanced solid tumors. Interim analyses of the HCC subgroup (*n* = 40) show that, at data cutoff, four patients out of 39 analyzed (10.3% 95% CI: 2.9–24.2) had achieved a partial response and response was ongoing in two out of four (50.0%) patients. Treatment related grade 3/4 AEs occurred in 20.0% of patients. The most common were elevated AST (7.5%) and elevated ALT (5.0%). Seven patients (17.5%) discontinued treatment due to an AE but none was related to treatment. There were no treatment related deaths [49]. Much like atezolizumab, durvalumab is being researched as combinational therapy, mostly with tremelimumab, a CTLA-4 inhibitor (see 2.2.2). Another awaited trial is the EMERALD-2 trial, which is a phase 3 trial combining durvalumab with bevacizumab.

#### 2.1.11. Avelumab

Avelumab is a human anti–PD-L1 IgG1 antibody. It is currently under investigation as monotherapy in patients with HCC after unsuccessful treatment with sorafenib in a phase 2 study. Results are expected soon. Furthermore, avelumab is being combined with axitinib, a TKI selective for VEGF, in a phase 1b study evaluating safety and efficacy [50]. In this study, patients received avelumab + axitinib until progression, unacceptable toxicity, or withdrawal. Assessment of preliminary data was performed after a minimum follow-up of 6 months based on the released study data set. In this analysis, 22 patients were treated with avelumab plus axitinib. ORR occurred in 13.6% (95% CI: 2.9–34.9) and 31.8% (95% CI: 13.9–54.9) when assessed by RECIST and mRECIST criteria, respectively. The most common grade 3 treatment-related AEs were hypertension in 11 (50.0%) patients and hand-foot syndrome in five (22.7%) patients. No grade 4/5 treatment related AEs were reported. So far, no patients discontinued treatment due to AEs. OS analyses were not yet conducted, as they were immature at the data cutoff. The authors concluded that the preliminary safety of avelumab + axitinib in HCC is manageable and consistent with the known safety profiles of avelumab and axitinib when administered as monotherapies. This study demonstrates antitumor activity of the combination in HCC.

### 2.2. CTLA-4 Pathway

The CTLA-4 protein is expressed on regulatory T cells (Tregs) and activation of this pathway takes place solely within lymph nodes. Here it regulates proliferation of activated lymphocytes. Unlike PD-1, CTLA-4 is expressed constitutively on Tregs where it is required for effector T cell inhibition through various mechanisms [51]. The idea behind this constant expression on Tregs is that CTLA-4 regulates physiologically unnecessary T cell activity and prevents excessive T cell immune responses. Under normal physiological conditions, CTLA-4 terminates T cell activity, when they are no longer needed.

In cancer however, CTLA-4 halts the activation and proliferation of activated T cells that are of great value in recognizing cancer antigens. CTLA-4 also promotes immunosuppression in the tumor microenvironment by enhancing Treg activity and differentiation as well as interfering with the function of dendritic cells [52]. This immunosuppressive mechanism in tumor environments can be counteracted by anti-CTLA-4-antibodies. Therapy with an anti-CTLA-4-antibody has as goal to release the brake on T cell activation in the lymph nodes. Since CTLA-4 is expressed at high levels by Tregs, one of the actions of anti-CTLA-4-antibodies may be to downregulate Tregs in the tumor microenvironment. A schematic overview of the above mentioned pathway is displayed in Figure 1. Currently approved and published studies of therapeutic agents of CTLA-4 are summarized in Table 1.

#### 2.2.1. Ipilimumab

Ipilimumab is a human monoclonal anti-CTLA-4-antibody. It was first studied as treatment for advanced melanoma [53]. In this trial, it showed clinical antitumor activity with minor AEs. In total 155 patients were included, of whom zero had CR and nine had PR (ORR of 6% (95% CI: 2.7–10.7)), with five PRs ongoing at the last tumor assessment. Treatment related AEs occurred in 84% of patients of whom 43 patients (28%) had grade 3/4 events. Serious AEs were reported for 83 patients (54%) and were considered drug related in 49 patients (32%). For HCC, ipilimumab has been tested in the CheckMate040 trial [31], with one experimental arm that included the evaluation of nivolumab (see Section 2.1.1) plus ipilimumab in a sub cohort of patients previously treated with sorafenib. Preliminary results of this trial showed an ORR of 31%, with a median duration of response of 17 months. Other trials that combine ipilimumab with other agents, mostly nivolumab, are currently on their way.

#### 2.2.2. Tremelimumab

Tremelimumab is a fully human monoclonal anti-CTLA-4-antibody and was first tested in HCC as checkpoint inhibitor in patients with chronic HCV infection [54]. This trial included 21 patients with HCV and HCC. Three patients discontinued the trial before the first response assessment. Among the remaining 17 patients that were evaluable for tumor response, ORR was 17.6%. No CRs were observed and three patients (17.6%) had a confirmed PR that lasted for 3.6, 9.2, and 15.8 months. Ten patients (58.8%) had an SD as the best response to treatment, accounting for a disease control rate of 76.4%. Treatment was overall well tolerated with few patients experiencing disabling AE.

In HCC the combination of durvalumab and tremelimumab has been researched in a phase 1/2 safety and efficacy trial [23]. In this trial, 40 patients with HCC were evaluable to assess the safety and tolerability of the durvalumab/tremelimumab combination. Out of 40 patients included, six (15%) patients had confirmed ORR, with all patients having PR. The ORR on the basis of confirmed and unconfirmed response was 20% (8 patients). Regarding the AEs: 20% had ≥1 grade ≥3 related AEs. Most common treatment related AEs: fatigue (20%), increased ALT (18%), pruritus (18%), and increased AST (15%). Most common grade ≥ 3 related AE was asymptomatic increased AST (10%).

As part of the 2020 ESMO World Congress on Gastrointestinal Cancer, findings from part three of the phase 2 ‘Study 22′ were presented. This part of the study was presented for 332 patients and tested several treatment regimens, revealing that the high-dose arm, a single dose of 300 mg of tremelimumab in combination with 1500 mg of durvalumab administered every 4 weeks, elicited significant clinical activity with a manageable safety profile in patients with unresectable HCC who reacted unfavorable on sorafenib. Regarding efficacy, the median OS was 18.7 months with the high-dose tremelimumab combination. The ORR was 24.0% in this arm. Regarding safety, treatment-related serious AEs including death occurred in 16.2% of patients treated with high-dose tremelimumab versus 10.9%, 24.6%, and 14.6% of patients in the durvalumab, tremelimumab, and low-dose tremelimumab arms, respectively.

Based on this data, the dosage of the highly awaited phase 3 clinical HIMALAYA trial was chosen. The HIMALAYA trial is a randomized, open-label, multicenter, global phase 3 study that aims to assess the efficacy of durvalumab monotherapy and the ‘high-dose regimen from the ‘study 22′ trial versus the standard-of-care medicine sorafenib. In total, a 1324 patients were enrolled into study assessment. Currently, the results of this trial are awaited.

An overview of the abovementioned phase 3 trials is given in Table 2.

## 3. Combination Therapy 

As described above, checkpoint inhibitors of PD-1, PD-L1 and CTLA-4 show promise in HCC treatment. Currently, another approach is being thoroughly researched. Combining several immune checkpoint inhibitors or combining an immune checkpoint inhibitor with a TKI, or curative/loco regional therapy is being researched in clinical trials. Recent trials in patients with melanoma showed increased results in combining checkpoint inhibitors [21]. These findings have inspired the use of combinational therapy in patients with HCC. Furthermore, combinational therapy has also already shown increased efficacy in various studies in patients with HCC, as described in chapter 2. Most recently many trials have started to combine immune checkpoint inhibitors with standard loco regional therapy such as resection, Radio Frequent Ablation (RFA), Microwave Ablation (MWA), Trans Arterial Chemoembolization (TACE), Selective Internal Radiotherapy (SIRT), and TKI’s. Currently, multiple phase 2 and 3 trials are ongoing in patients with HCC. Clinical phase 3 trials using combinational strategies are summarized in Table 2. Amongst many phase 3 trials, plenty of phase 1 and 2 trials are currently ongoing or are on their way. Table 3 gives a total overview of all ongoing and upcoming trials regarding immune checkpoint inhibition therapy in patients with HCC. It deserves the notion that almost all clinical trials with immune checkpoint inhibitors that are being conducted, or are started, are being done so with PD-1 and/or PD-L1 immune checkpoint inhibition. It is clear that researchers see most clinical future in these treatment strategies.

## 4. Toxicity

Although many studies have reported better tolerability of immune checkpoint inhibitors in various forms of solid cancers, patients with HCC might react differently to immunotherapy. This has to do with the fact that HCC is almost always a result of underlying liver cirrhosis and liver disease where liver function is compromised. This might result into overreaction or more pronounced (liver-related) toxicity in patients with HCC when treated with immune checkpoint inhibitors. Overall grade 3/4 AEs reported in published studies of immune checkpoint monotherapy in patients with HCC (Table 1) varies from 12–25%. Depending on the therapeutic agent, AEs can vary from hypertension (atezolizumab) to fatigue (tislelizumab and tremelimumab) to elevated AST (camrelizumab). Postow et al. has given a comprehensive overview in possible immune-related AEs that occur in treatment with immune checkpoint inhibition [20].

For patients with HCC, the most pronounced immune-related hepatotoxicity AEs in the CheckMate040 and Keynote-224 was elevation in liver enzymes (AST and ALT). This only occurred in the minority, and if so, was usually mild (grade 1/2) (15–20% overall, and less than 10% grade 3/4 AEs) [31,33]. A recent subgroup analyses of patients with CP B7-8 in the CheckMate040 study demonstrated similar safety profiles with no more treatment discontinuation for patients when compared to the initial dose-escalation phase of the study that had mostly CP A patients [55]. In the phase 1a/1b trial of tislelizumab four different types of cancer were treated. AEs were roughly the same for all types of cancer with the only outlier being fatal acute hepatitis in a patient with HCC with rapidly progressing disease [35]. When comparing these AEs to patients treated for advanced melanoma, toxicity profiles were about the same, occurring in ±20% of patients treated with toripalimab [42].

## 5. Response Predictors 

As described above, immune checkpoint inhibitors have great potential when it comes to treating patients with HCC. However, there is a large heterogeneity in response between patients. This possibly has to do with the fact that not all HCC-tumors express the abovementioned proteins in the same frequency or intensity. This can also explain why the results from several immune checkpoint inhibitor monotherapy studies show disappointing results. Then again, a high PD-L1 expression on tumor cells was identified as a predictor for HCC recurrence in patients. Moreover, a high PD-L1 expression on tumors was significantly associated with HCC tumor aggressiveness. Interestingly enough, the PD-L1 expression on tumors may be predictive for anti-PD-1 and/or anti-PD-L1 treatment [56]. Therefore, it would be ideal to have objective biomarkers that can identify which patients are more likely to respond to immune checkpoint inhibition. So far, expression of PD-L1 on tumor cells has been identified as potential biomarker. It showed correlation between expression of PD-L1 and ORR in patients with a variety of cancers [57]. However, in patients with HCC, no such correlation was found in the CheckMate040 trial [31]. Data from a more recent study suggest that serum PD-1 and PD-L1 are independent prognostic factors for both disease-free survival (DFS) and OS in patients with HCC [58]. Another challenge for developing or identifying a predictive biomarker lies in the tendency to combine immune checkpoint inhibitors with other treatment modalities. The most potential for treatment success currently lies in combining therapeutic options, such as multiple immune checkpoint inhibitors or addition of an anti-VEGF antibodies. This makes it hard to identify an all-round biomarker that can accurately predict response over the full range of treatment options within the immune checkpoint inhibition armamentarium. Currently, investigation in other biomarkers such as tumor mutational burden, gut microbiome and other genetic profiles is undergoing and awaits [59]. Based on experience with biomarkers in other types of solid cancers, further investigation is directed. For now: no clinical use in biomarkers in patients with HCC is validated yet.

## 6. Conclusions

In the last few years, immunotherapy has permanently changed the field of systemic therapy in patients with advanced HCC. The results from various phase 2 and 3 studies show great results in the use of PD-1, PD-L1 and CTLA-4 checkpoint inhibitors. Even though the results from phase 3 studies investigating the efficacy of immune checkpoint inhibitors as monotherapy versus placebo or TKI’s in the first or second-line were somewhat disappointing, it nevertheless changed the playing field of anti-cancer treatment and inspired further investigation into this treatment modality. With this inspiration, combinational therapies (with immune checkpoint inhibitors and/or standard of care treatment options) were explored and already show great promise towards becoming the new cornerstone of HCC treatment. One of the challenges in making conclusions in terms of HCC treatment is that most studies that have been conducted so far, such as the CheckMate040 and KEYNOTE study, are not randomized studies designed to assess efficacy in HCC specifically. Therefore, further studies specifically designed for patients with HCC are advised. In the studies that have been HCC specific, or had specified subgroups, toxicity and tolerability was well manageable over the full scope of use in patients with HCC. This field is rapidly evolving and many discoveries are yet to be made. Nonetheless, the future perspective for patients with incurable HCC has taken a turn for the better. Seen that the amount of clinical trials is rapidly growing, especially in the combination of PD-1 and PD-L1 antibodies, the next challenge will certainly be finding the right sequence and timing of treatment with immunotherapeutic agents and what to combine them with. Possibly, biomarkers and other patient or tumor characteristics will help physicians make a personalized choice, acuminated to patient needs and wishes, achieving the best possible care patients can get.

## Figures and Tables

**Table 1:** Publicized studies of approved immune checkpoint inhibitors, monotherapy and combinational therapy, in HCC, displaying results and adverse events.

Pathway	Drug	Study Name/Phase	Setting	*n*	ORR	Result	Grade 3/4 AEs	Ref
**PD-1**	Nivolumab	CheckMate 040 Phase 1/2 CheckMate 459 Phase 3	First-line First-line	214 743	20% 15%	Positive Negative	25% 22%	[31] [32]
	Pembrolizumab	Keynote-224 Phase 2 Keynote-240 Phase 3	Non-comparative Second-line	104 413	18% 18%	Positive Negative	24% 19%	[33] [34]
	Tislelizumab	Phase 1a/1b	First in-human trial	207	12%	Positive	18%	[35]
	Camrelizumab	Phase 2	Second-line	217	15%	Positive	22%	[37]
	Sintilimab	Phase 1b	Second-line	50	26%	Positive	12%	[40]
	Spartalizumab	Phase 1	First in-human trial	58	3%	N/A	N/A	[46]
**PD-L1**	Atezolizumab	GO30140 Phase 1b IMbrave150 Phase 3	Monotherapy And + bevacizumab in advanced HCC + bevacizumab in first-line treatment	59 104 501	17% 36% N/A	Positive Positive Positive	5% 42% 57%	[44] [44] [45]
	Durvalumab	Phase 1/2	HCC subgroup	50	10%	Positive	20%	[49]
	Avelumab	Phase 1b	+axitinib	22	14%	Positive	73%	[50]
**CTLA-4**	Ipilimumab	Checkmate 040 Phase 1/2	+nivolumab	N/A	31%	Positive	N/A	[31]
	Tremelimumab	Study-22 Phase 2	Dose finding study	332	24%	Positive	16%	[47]

Legend: AEs: Adverse Events; HCC: Hepatocellular Carcinoma; ORR: Objective Response Rate; VEGF: Vascular Endothelial Growth Factor.

**Table 2:** Currently ongoing phase 3 trials with immune checkpoint inhibitors as monotherapy or in combinational setting in patients with HCC.

Agent	Combining Modality	Mechanism	Trial Name/Code	*n*	Setting
Nivolumab	Ipilimumab	CTLA-4	CheckMate040	148	Advanced HCC (first-line)
	Ipilimumab	CTLA-4	CheckMate9DW	1084	Advanced HCC (first-line)
	Ipilimumab	CTLA-4	CheckMate74W	765	Adjuvant to TACE
			CheckMate9DX	530	Adjuvant after resection in patients with high risk of recurrence
	TACE	Chemoembolization	NCT04268888	522	Adjuvant
Pembrolizumab	Lenvatinib + TACE	TKI + chemoembolization	LEAP-012	950	Adjuvant
	Lenvatinib	TKI	LEAP-002		
			KEYNOTE-937	950	Adjuvant to complete radiological response after resection or ablation
	BSC		KEYNOTE-394	750	Advanced HCC
Tislelizumab			Rationale 301	450	Advanced HCC (first-line)
Camrelizumab	FOLFOX4	Chemo	NCT03605706	396	Advanced HCC (first-line)
	Apatinib	VEGFR2	NCT03764293	510	Advanced HCC (first-line)
Sintilimab	IBI305	VEGF	ORIENT-32	566	Advanced HCC (first-line)
	SBRT	Radiation	NCT04167293	116	Advanced HCC with portal vein invasion
Toripalimab			JUPITER 04	402	vs placebo after resection
	Lenvatinib	TKI	NCT04523493	486	vs placebo in advanced HCC
			NCT03949231	200	Toripalimab via hepatic artery/vein infusion in BCLC stage C HCC
**Atezolizumab**	Cabozantinib	TKI	COSMIC-312	740	Advanced HCC (first-line)
	Bevacizumab	VEGF	AMETHISTA	150	Advanced HCC (first-line)
	Bevacizumab	VEGF	IMBrave 150	480	Advanced HCC (first-line)
	Bevacizumab	VEGF	IMBrave 050	662	Adjuvant after resection in patients with high risk of recurrence
**Durvalumab**	Tremelimumab	CTLA-4	HIMALAYA	1324	Advanced HCC (first-line)
	Bevacizumab + TACE	VEGF + chemoembolization	EMERALD-1	600	vs placebo in loco regional HCC
	Bevacizumab + TACE + Tremelimumab	VEGF + chemoembolization + CTLA-4	EMERALD-2	888	(Neo-)adjuvant to TACE in advanced HCC

Legend: BCLC: Barcelona Clinic Liver Cancer; BSC: Best Supportive Care; HCC: Hepatocellular Carcinoma; TACE: Trans Arterial Chemoembolization; TKI: Tyrosine Kinase Inhibitor; VEGF: Vascular Endothelial Growth Factor.

**Table 3:** Ongoing/coming trials with immune checkpoint inhibitors in the treatment of HCC (as per 1-10-2020).

Agent	Combined With	Mechanism	Setting	*n*	Phase R(arm)/NR	Expected Finishing Date	ClinicalTrial.gov
Nivolumab	P1101	Biological	Advanced HCC	72	1/2 R(3)	31-07-2023	NCT04233840
Nivolumab	Pexa Vec	Oncolytic virus	Advanced HCC	30	1/2 NR	01-09-2020	NCT03071094
Nivolumab	Bevacizumab	VEGF	Advanced HCC	60	2 R(3)	31-10-2021	NCT04393220
Nivolumab	+/− Ipilimumab	CTLA-4	Neo-adjuvant prior to resection	30	2 R(2)	30-09-2022	NCT03222076
Nivolumab	TACE	Chemo	Adjuvant	49	2 NR	01-06-2023	NCT03572582
Nivolumab	Regorafenib	TKI	Advanced HCC progressive under sorafenib	60	2 NR	01-12-2022	NCT04170556
Nivolumab	Regorafenib	TKI	Advanced HCC	42	2 NR	30-05-2023	NCT04310709
Nivolumab	SIRT	Radiation	Adjuvant	27	1	01-07-2023	NCT02837029
Nivolumab	SF1126	mTOR inhibitor	Advanced HCC	14	1	01-10-2022	NCT03059147
Nivolumab	Lenvatinib	TKI	Advanced HCC	30	1	01-06-2021	NCT03418922
Nivolumab	Lenvatinib	TKI	Advanced HCC	50	2 NR	01-10-2021	NCT03841201
Nivolumab	SIRT	Radiation	Advanced HCC	40	2 NR	01-12-2019	NCT03033446
Nivolumab	BMS-986253/Cabiralizumab	Anti- IL-8/CSF1R	Advanced HCC	74	2 R(3)	05-08-2024	NCT04050462
Nivolumab	BMS-986205	IDO1 inhibitor	Advanced HCC (first/second line)	23	1/2 NR	01-06-2022	NCT03695250
Nivolumab	Cabozantinib	TKI	Neo-adjuvant prior to resection	15	1	01-03-2022	NCT03299946
Nivolumab	Ipilimumab	CTLA-4	Neo-adjuvant prior to resection	40	2 NR	31-12-2022	NCT03510871
Nivolumab	Ipilimumab	CTLA-4	Neo-adjuvant prior to resection	32	2 NR	01-09-2022	NCT03682276
Nivolumab	Ipilimumab	CTLA-4	Neo-adjuvant prior to TACE	35	2 NR	01-09-2024	NCT04472767
Nivolumab	GT90001	Anti-ALK-1	Advanced HCC + metastasis	20	1/2 NR	25-09-2025	NCT03893695
Nivolumab	Ipilimumab	CTLA-4	Adjuvant after SBRT	50	1	01-08-2022	NCT03203304
Nivolumab	TACE	Chemo	Adjuvant	522	2/3 R(2)	01-06-2026	NCT04268888
Nivolumab	Relatlimab	Anti-LAG-3	Advanced HCC after TKI	250	2 (R3)	16-09-2023	NCT04567615
Nivolumab	ABX196	iNKT activation	Advanced HCC	48	1/2 NR	30-06-2021	NCT03897543
Nivolumab	Sorafenib	TKI	Advanced HCC +/− metastasis	40	2 NR	30-09-2022	NCT03439891
Pembrolizumab			Advanced HCC	29	2 NR	01-11-2022	NCT02658019
Pembrolizumab			Advanced HCC (after sorafenib)	60	2 NR	01-12-2020	NCT03163992
Pembrolizumab			Neo-adjuvant prior to resection/ablation	50	2 NR	31-10-2020	NCT03337841
Pembrolizumab	Sorafenib	TKI	Advanced HCC +/− metastasis	27	1/2 NR	13-09-2021	NCT03211416
Pembrolizumab	Lenvatinib	TKI	Advanced HCC	104	1	31-08-2021	NCT03006926
Pembrolizumab	Cabozantinib	TKI	Advanced HCC (first-line)	29	2 NR	13-09-2024	NCT04442581
Pembrolizumab	Regorafenib	TKI	Advanced HCC (first-line)	57	1	05-10-2022	NCT03347292
Pembrolizumab	Bavatuximab	Anti- phospholipids	Advanced HCC	34	2 NR	01-04-2022	NCT03519997
Pembrolizumab	TACE/MWA/RFA	Chemo/ablation	Adjuvant	30	2 NR	01-09-2023	NCT03753659
Pembrolizumab	TACE	Chemo	Adjuvant	26	1/2 NR	31-12-2020	NCT03397654
Pembrolizumab	Lenvatinib	TKI	Neo-adjuvant in HCC beyond Milan	192	N/A	30-12-2024	NCT04425226
Pembrolizumab	SBRT	Radiation	Advanced HCC progressive under sorafenib	30	2 NR	02-04-2022	NCT03316872
Pembrolizumab	SIRT	Radiation	Advanced HCC	30	1	01-01-2021	NCT03099564
Tislelizumab	Regorafenib	TKI	Advanced HCC (first-line)	125	2 R(2)	01-03-2025	NCT04183088
Tislelizumab	Lenvatinib	TKI	Advanced HCC +/− metastasis	66	2 NR	01-12-2022	NCT04401800
Tislelizumab			Advanced HCC (second-line)	249	2 NR	01-09-2021	NCT03419897
Camrelizumab			Advanced HCC	1000	N/A	01-03-2023	NCT04487704
Camrelizumab	Apatinib	VEGFR2	Advanced HCC	40	2 NR	01-10-2020	NCT04014101
Camrelizumab	Apatinib	VEGFR2	Advanced HCC	190	2 NR	30-12-2019	NCT03463876
Camrelizumab	Apatinib	VEGFR2	After radical hepatectomy	45	N/A	01-03-2020	NCT03722875
Camrelizumab vs. TACE			After resection + PVTT	40	N/A	31-01-2020	NCT03914352
Camrelizumab vs. TACE	Apatinib + TACE	VEGFR2 + Chemo	Advanced HCC	188	2 R(2)	01-09-2023	NCT04559607
Camrelizumab	SBRT/IMRT	Radiation	Adjuvant	39	2 NR	30-07-2020	NCT04193696
Camrelizumab	Apatinib	VEGFR2	Advanced HCC	30	2 NR	31-01-2021	NCT03793725
Camrelizumab vs. HAI	Apatinib	VEGFR2	After resection + high risk recurrence	200	2 R(2)	28-02-2023	NCT03839550
Camrelizumab	Apatinib	VEGFR2	Perioperative	20	2 NR	01-12-2021	NCT04297202
Camrelizumab			Recurrent HCC after LTx	20	1	01-07-2023	NCT04564313
Camrelizumab	Apatinib + RT	VEGFR-2 + Radiation	Advanced HCC with metastasis	27	2 NR	01-08-2022	NCT04523662
Camrelizumab	Apatinib + TACE + FOLFOX	VEGFR2 + Chemo	Adjuvant	56	2 NR	31-12-2023	NCT04479527
Camrelizumab	Lenvatinib	TKI	Advanced HCC (first-line)	53	1/2 NR	01-08-2023	NCT04443309
Camrelizumab	RFA	Ablation	Advanced HCC	120	2 NR	30-12-2026	NCT04150744
Camrelizumab	Apatinib + HAI	VEGFR2 + chemo	BCLC-C HCC	84	2 NR	31-12-2025	NCT04191889
Camrelizumab	Apatinib	VEGFR2	Neo-adjuvant prior to LTx	120	1/2 NR	31-12-2021	NCT04035876
Sintilimab	SBRT	Radiation	Adjuvant	30	2 NR	28-02-2022	NCT03857815
Sintilimab	IBI305	VEGF	Advanced HCC	45	1	11-11-2021	NCT04072679
Sintilimab	Ipilimumab	CTLA-4	Advanced HCC	47	1b	01-04-2023	NCT04401813
Sintilimab	TAI	Chemo	Adjuvant	40	2 NR	25-03-2022	NCT03869034
Sintilimab	TACE	Chemo	Adjuvant	25	2 NR	10-05-2023	NCT04297280
Sintilimab	Apatinib + Capecitabine	VEGFR2 + chemo	Advanced HCC	46	2 NR	01-06-2022	NCT04411706
Sintilimab	TACE	Chemo	Neo-adjuvant in HCC A/B beyond Milan	61	2 NR	30-05-2022	NCT04174781
Sintilimab	Radiotherapy	Radiation	Adjuvant in HCC + PVTT	20	1	31-12-2021	NCT04104074
Sintilimab	Lenvatinib	TKI	Advanced HCC	56	2 NR	30-08-2024	NCT04042805
Sintilimab	Anlotinib	TKI	Advanced HCC	20	2 NR	30-12-2021	NCT04052152
Sintilimab	TACE/MWA	Chemo/ablation	Advanced HCC	45	1	30-09-2021	NCT04220944
Sintilimab	SBRT	Radiation	Advanced HCC + metastasis	84	2 R(2)	01-07-2023	NCT04547452
Toripalimab	After LTx	Transplant	Adjuvant	20	1	31-10-2022	NCT03966209
Toripalimab	ATG-008	mTOR inhibitor	Advanced HCC	38	N/A	17-03-2022	NCT04337463
Toripalimab	Lenvatinib	TKI	Advanced HCC	76	2 NR	01-04-2023	NCT04368078
Toripalimab	TAI	Chemo	Advanced HCC	65	2 NR	02-03-2021	NCT03851939
Toripalimab	SBRT	Radiation	Advanced HCC + PVTT	30	2 NR	01-01-2021	NCT04169399
Toripalimab	Lenvatinib, HAIC	TKI, chemo	Advanced HCC	36	2 NR	01-10-2020	NCT04044313
Toripalimab	HAIC	Chemo	Advanced HCC + PVTT (first-line)	60	2 R(2)	20-10-2020	NCT04135690
Toripalimab	Chemo + lenvatinib	Chemo + TKI	Advanced HCC + metastasis	25	2 NR	01-12-2020	NCT04170179
Toripalimab	Sorafenib	TKI	Advanced HCC + PVTT	39	1/2 NR	01-10-2021	NCT04069949
Atezolizumab vs. SIRT	Bevacizumab	VEGF	Advanced HCC	128	2 R(2)	01-01-2024	NCT04541173
Atezolizumab	Bevacizumab + TACE	VEGF + chemo	(Neo-)Adjuvant prior to/following TACE in Advanced HCC	106	2 R(2)	01-03-2025	NCT04224636
Atezolizumab	Bevacizumab	VEGF	Advanced HCC + HBV	48	2 NR	30-06-2022	NCT04180072
Spartalizumab	+/−Capmatinib	MET-inhibitor	Advanced HCC	90	1b/2	20-10-2020	NCT02795429
Genolimzumab	Bozotinib	c-Met inhibitor	Locally advanced or metastatic HCC	119	1/2	15-12-2020	NCT03655613
Durvalumab	+/−Tremelimumab	Hypo-fractionated Radiotherapy	After initial treatment with anti-PD-1	30	2 NR	01-08-2024	NCT04430452
Durvalumab			Advanced HCC with active HBV	43	2 NR	31-12-2022	NCT04294498
Durvalumab + Tremelimumab	TACE/RFA/Cryo	Chemo/Ablation	Advanced HCC	90	2 NR	31-12-2021	NCT02821754
Durvalumab + Tremelimumab	SIRT/TACE	Radiation/Chemo	Advanced HCC	84	2 R(2)	30-09-2024	NCT04522544
Durvalumab	Tivozanib	TKI	Advanced HCC (first-line)	42	1/2 NR	01-08-2022	NCT03970616
Durvalumab	TACE + ablation	Chemo + ablation	Advanced HCC	30	N/A	01-10-2024	NCT04517227
Durvalumab	Tremelimumab + radiation	CTLA-4 + radiation	Advanced HCC	70	2 NR	31-10-2025	NCT03482102
Durvalumab	Tremelimumab	CTLA-4	Advanced HCC	433	2 R(5)	31-12-2021	NCT02519348
Durvalumab	Bevacizumab	VEGF	Advanced HCC	433	2 R(5)	31-12-2021	NCT02519348
Durvalumab			Advanced HCC	433	2 R(5)	31-12-2021	NCT02519348
Durvalumab	Tremelimumab + TACE	CTLA-4	Adjuvant after TACE	30	2 NR	01-11-2020	NCT03638141
Durvalumab	SIRT	Radiation	Adjuvant after SIRT	24	1/2 NR	30-12-2021	NCT04124991
Durvalumab	Lenvatinib	TKI	Advanced HCC + metastasis	20	N/A	31-12-2025	NCT04443322
Durvalumab	TACE + Bevacizumab + Tremelimumab	Chemo + VEGF + CTLA-4	(Neo-)Adjuvant with TACE	22	2 NR	31-12-2022	NCT03937830
Avelumab			Advanced HCC (second-line)	30	2 NR	31-03-2020	NCT03389126
Tremelimumab			Advanced HCC	433	2 R(5)	31-12-2021	NCT02519348

Legend: BCLC: Barcelona Clinic Liver Cancer; BSC: Best Supportive Care; HAI(C): Hepatic Artery Infusion (Chemo); HCC: Hepatocellular Carcinoma; HVI: Hepatic Vein Infusion; LTx: Liver Transplant; MWA: Microwave Ablation; NR: Not Randomized; R: Randomized; RFA: Radiofrequent Ablation; RT: Radiation Therapy; SBRT: Stereotactic Body Radiation Therapy; SIRT: Selective Internal Radiation Therapy; TACE: Trans Arterial Chemoembolization; TAI: Trans Arterial Infusion; TKI: Tyrosine Kinase Inhibitor; VEGF: Vascular Endothelial Growth Factor.
